# Chronology of Deep Nodes in the Neotropical Primate Phylogeny: Insights from Mitochondrial Genomes

**DOI:** 10.1371/journal.pone.0051699

**Published:** 2012-12-14

**Authors:** Carlos G. Schrago, Albert N. Menezes, Miguel A. M. Moreira, Alcides Pissinatti, Hector N. Seuánez

**Affiliations:** 1 Departamento de Genética, Universidade Federal do Rio de Janeiro, Rio de Janeiro, Rio de Janeiro, Brazil; 2 Programa de Genética, Instituto Nacional do Câncer, Rio de Janeiro, Rio de Janeiro, Brazil; 3 Centro de Primatologia do Rio de Janeiro, Rio de Janeiro, Brazil; Texas A&M University, United States of America

## Abstract

The evolution of Neotropical Primates (NP) is permeated by factors associated with the pattern of diversification and the biogeography of the major lineages. These questions can be better understood by providing a robust estimate of the chronological scenario of NP evolution, a reason why molecular dating methods have been widely applied. One aspect of especial interest is the timing of diversification of the major NP lineages (pitheciids, atelids and cebids), which may have resulted from rapid episodes of adaptive radiation, a question that requires NP divergence time estimates with accurate statistical certainty. In this study, we evaluated the primate timescale focused on the age of nodes of NP radiation. We investigated the performance of complete primate mitochondrial genomes as traditional molecular markers of primate evolution and further including original mitochondrial data from the endangered muriqui, *Brachyteles arachnoides* (Accession No. JX262672). Comparisons of the age estimates at NP nodes based on mitochondrial genomes with those obtained from a nuclear supermatrix showed similar degrees of uncertainty. Further molecular data and more informative calibration priors are required for a more precise understanding of the early NP diversification.

## Introduction

Although the diversification of Neotropical Primates (NP; Primates, Anthropoidea, Platyrrhini) has recently been a subject of detailed analyses [1], [2], [3], [4] several aspects of the evolutionary history of this group still remains poorly understood. This is the case of how NP emerged and the geoclimatic scenario that enabled the ancestral old world anthropoid stock to reach the New World island-continent in the Eocene/Oligocene transition, a setting requiring a well-established timescale [5]. Similarly, the different propositions on the diversification patterns of the crown Platyrrhini clade and the phylogenetic affinities of NP fossils from Miocene deposits from Argentina are tightly dependent on the timing of NP evolution [6].

Furthermore, the conspicuous spatial distribution of some NP lineages has triggered the investigation of the underlying ecological and geological factors operating during their biogeographic history. This is particularly noticeable in lineages comprising Amazonian and non-Amazonian representatives, like the (*Mico*, *Cebuella*)/*Callithrix* sister genera [7], the gracile and robust *Cebus* groups [8], and *Callicebus* species [9]. The genus *Brachyteles* is also of biogeographic interest because its two extant species are distributed in the Atlantic forest contrary to other representatives of the tribe Atelini which inhabit the Amazonian forest.

Phylogenomic analyses have been crucial for more accurate age estimations of NP nodes in topologies based on high number of genes for inferring NP timescales [10], while an increasing number of NP genome projects is presently underway [11]. Thus, the amount of molecular data available in the years to come will likely reduce the inherent stochastic error of divergence time inferences.

Mitochondrial genomes comprise the most abundant genomic material per species for studying primate evolution. At present, while only some 12 nuclear primate genomes have been partially or completely analyzed, more than 50 complete primate mitochondrial genomes are publicly available. It is therefore relevant to investigate whether timescales based on this genomic component are equivalent to those inferred from nuclear DNA supermatrices.

In this study, we have inferred the primate timescale based on mitochondrial genomes, with special emphasis on NP diversification, a controversial point in NP systematics. Studies conducted to present have reported that the diversification of pitheciids, atelids and cebids has been a rapid event, an evolutionary pattern that could only be understood by estimating divergence times with minimum uncertainty. In order to increase taxonomic sampling of NP nodes, we sequenced the complete mitochondrial genome of the endangered muriqui, *Brachyteles arachnoides*. Our findings indicated that divergence time estimates inferred from mitochondrial genomes were similar to estimates with nuclear gene supermatrices, and that the amount of effective informative data of mitochondrial genomes was equivalent to the one provided by nuclear supermatrices.

## Materials and Methods

### Collection of Samples and Ethical Considerations

Peripheral blood samples were obtained from a captive, male *Brachyteles arachnoides* (CPRJ2506) kept in the Centro de Primatologia do Rio de Janeiro (CPRJ-INEA). Samples used in this study were part of the blood samples regularly collected for checkups and control of captive animals. Sample collection was carried out following the national guidelines and provisions of IBAMA (Instituto Brasileiro do Meio Ambiente e dos Recursos Naturais Renováveis, Brazil; permanent license number 11375-1). The granting of this licence by IBAMA followed approval by its Ethics Committee. The Centro de Primatologia do Rio de Janeiro is located 100 km northeast of the city of Rio de Janeiro, in a protected forest area of the Serra dos Órgãos mountain range. The Centro is not open to visitors. In this facility, animals are housed in groups in outdoor enclosures consisting of wire mesh, being exposed to the Atlantic Forest conditions such as sounds, temperature, and rainfall. The *Brachyteles* enclosures measure 16.0 m×6.0 m×6.0 m high with an additional area of 2 m×6 m×3 m high for feeding. Food and fresh water were provided twice a day. The diet consisted of bread, bananas, eggs, raisins, meat, and various commercially prepared protein supplements.

Genomic DNA was extracted with QIAamp**®** DNA Mini and Blood Mini kit (Quiagen**®**). DNA was quantified with a NanoDrop spectrophotometer and its quality and integrity were checked by electrophoresis in 1% agarose gels. Genomic DNA was fragmented with an Invitrogen nebulizer under 40 psi for 40 seconds in TE buffer and 25% glicerol. Following the observation of 300–400 bp fragments by electrophoresis, DNA was quantified using a Qubit 2.0 fluorometer. Library preparation followed Illumina Sequencing Workflow protocols for the HiSeq2000 platform. DNA containing 400–500 bp fragments, checked with Agilent Bioanalyzer 2100 DNA using a high sensitivity DNA chip, was subsequently tested by qPCR using a Library Quantification kit for library validation (KAPA – KK4824). DNA Cluster generation was prepared for 2 lanes of a PE Flowcell v.3 following the manufacturer’s protocol. A 75×75 run was carried out in an Illumina HiSeq2000 platform. Cycles registered 84.4% base calls with Q30 quality score. The mitochondrial genome was assembled using the CLC Genomics Workbench 5.1 with default settings. Genome assembly was performed using published Platyrrhini mitochondrial genomes as references. We also restricted minimum contig length to >13,000 bp to speed up the process. On average, coverage of the mitochondrial genome was 165X.

Open reading frames were manually annotated.

### Evolutionary Analysis

Complete mitochondrial genomes of 42 selected primates were downloaded from GenBank (Table 1). We have independently aligned the 13 protein coding mitochondrial genes, which were subsequently concatenated in an 11,672 bp supermatrix. The supermatrix was partitioned into codon positions. Evolutionary model choice was implemented with HyPhy using the likelihood ratio test [12]. Bayesian phylogenetic inference was conducted with MrBayes 3.2 [13] allowing for independent evolutionary parameters of the GTR+G model (nst = 6 and rates = gamma) for each partition, i.e., codon position. In MrBayes, Markov chain Monte Carlo (MCMC) was run independently 3 times (nruns = 3) with one cold and two hot chains (nchains = 3). In each run, chains were sampled every 100^th^ cycle until 100,000 topologies were obtained. When topologies were summarized, the first 10,000 trees were discarded as burn-in. Convergence of the MCMC run was diagnosed by the potential scale reduction factor, which approached 1.00 for all parameters. We have also conducted maximum likelihood (ML) phylogenetic inference in PhyML 3 [14] using the GTR+G model. Tree topology search was implemented using both the nearest neighbor interchange and subtree pruning and regrafting methods. In PhyML, we used the approximate likelihood ratio test for obtaining statistical support of the clades [15].

**Table 1:** Mitochondrial genomes used in this study.

Species name			Accession number
Strepsirrhini			
		*Otolemur crassicaudatus*	NC_012762
		*Lemur catta*	NC_004025
			
Tarsiidae			
		*Tarsius bancanus*	NC_002811
		*Tarsius syrichta*	NC_012774
			
Catarrhini	Cercopithecoidea		
		*Chlorocebus aethiops*	NC_007009
		*Chlorocebus pygerythrus*	NC_009747
		*Chlorocebus sabaeus*	NC_008066
		*Chlorocebus tantalus*	NC_009748
		*Colobus guereza*	NC_006901
		*Macaca fascicularis*	NC_012670
		*Macaca mulatta*	NC_005943
		*Macaca sylvanus*	NC_002764
		*Macaca thibetana*	NC_011519
		*Nasalis larvatus*	NC_008216
		*Papio hamadryas*	NC_001992
		*Piliocolobus badius*	NC_008219
		*Presbytis melalophos*	NC_008217
		*Pygathrix nemaeus*	NC_008220
		*Rhinopithecus avunculus*	NC_015485
		*Rhinopithecus bieti*	NC_015486
		*Rhinopithecus roxellana*	NC_008218
		*Semnopithecus entellus*	NC_008215
		*Trachypithecus obscurus*	NC_006900
	Hominoidea		
		*Gorilla gorilla*	NC_001645
		*Homo sapiens*	NC_012920
		*Hylobates agilis*	NC_014042
		*Hylobates lar*	NC_002082
		*Hylobates pileatus*	NC_014045
		*Nomascus siki*	NC_014051
		*Pan paniscus*	NC_001644
		*Pan troglodytes*	NC_001643
		*Pongo abelii*	NC_002083
		*Pongo pygmaeus*	NC_001646
		*Symphalangus syndactylus*	NC_014047
			
Platyrrhini	Cebidae		
		*Aotus lemurinus*	FJ785421
		*Cebus apella*	NC_016666
		*Cebus albifrons*	NC_002763
		*Saimiri sciureus*	NC_012775
		*Saguinus oedipus*	FJ785424
	Atelidae		
		*Ateles belzebuth*	FJ785422
		*Brachyteles arachnoides*	JX262672
	Pitheciidae	*Callicebus donacophilus*	FJ785423

Estimation of divergence time was conducted with the MCMCTree program of the PAML 4.5 package [16]. This program implements the method of Rannala and Yang [17] which models the evolution of substitution rates along branches using a log-normal distribution in which the mean rate of a branch is independent of the rate of its ancestral branch (clock = 2). To approximate the posterior density of divergence times, the MCMC algorithm was run for 50,000,000 generations and sampled every 100^th^ cycle; 10,000 generations were discarded as burn-in. Prior distribution settings for node ages (birth and death model) and evolutionary rates (gamma distribution) were as follows: BDparas = 1 1 0.1; rgene_gamma = 2 2; sigma2_gamma = 1 10. Convergence of the MCMC run was diagnosed by the potential scale reduction factor, which approached 1.00 for all parameters and by calculating effective sample sizes, which were greater than 200 for all parameters.

Calibration data used to constrain the prior distribution of age nodes was carried out as follows. The age of the basal diversification of the Primates was constrained to be older than 55.6 Ma, based on the fossil record of *Altiatlasius* sp. [18]. The split between lemuroid and lorisoid prosiminans was modeled by a uniform distribution with minimum and maximum limits at 33.7 Ma (Mega annum, or million years ago) and 55.6 Ma respectively [19]. The minimum value of the basal Anthropoid diversification was set at 33.7 Ma, based on the age of *Catopithecus* sp., a stem Catarrhine primate [20]. The Hominoid/Cercopithecoid split was calibrated by a uniform distribution from 34.0 Ma to 23.5 Ma based on the age of *Proconsul* sp. [21]. The separation of the *Pongo* lineage to the other great apes was constrained by a uniform prior from 33.7 Ma to 11.2 Ma [22]. The *Gorilla* split was enforced to be older than 7.25 Ma and, finally, the *Homo*/*Pan* divergence was constrained between 5.7 Ma and 10.0 Ma [23].

With the purpose of investigating whether the information contained in mitochondrial genomes approached its theoretical limit, we plotted the width of the 95% credibility interval (the highest probability density, HPD, interval) of posterior densities of age estimates against their mean values. Theoretically, when alignment size approaches infinity, we would expect a perfect linear relationship between node age (*t*) and credibility intervals (*w*) [17], [24]. Thus, by fitting a regression line on the *w* × *t* plot, we calculated the degree of uncertainty of estimates associated with sequence length, i.e., the stochastic error. If the fit is well supported, further increase in precision of estimates is only possible by the adoption of more informative calibration priors. For the sake of comparison, we also measured the fit of the *w* × *t* regression line for estimates of the recent comprehensive analysis of Perelman et al. [10] who used a 34,941 bp supermatrix of nuclear genes with 190 terminals.

## Results

The *Brachyteles arachnoides* mitochondrial genome was 16,491 bp long (GenBank JX262672); gene order being the same as in mammals (Table 2) and with base frequencies corresponding to values recorded for platyrrhines: *f*_A_ = 31.4%, *f*_C_ = 27.5%, *f*_G_ = 12.5% and *f*_T_ = 28.6%.

**Table 2:** List of genes of mtDNA of *Brachyteles arachnoids.*

Genes	Start	Stop	Strand	Length
*MT-RNR2* *	15,804	783	+	1,471
*MT-TL2*	784	858	+	75
*MT-ND1*	861	1,816	+	956
*MT-TI*	1,817	1,885	+	69
*MT-TQ*	1,883	1,954	−	72
*MT-TM*	1,958	2,025	+	68
*MT-ND2*	2,028	3,065	+	1,038
*MT-TW*	3,067	3,133	+	67
*MT-TA*	3,142	3,210	−	69
*MT-TN*	3,212	3,284	−	73
*MT-TC*	3,318	3,384	−	67
*MT-TY*	3,384	3,448	−	65
*MT-CO1*	3,456	4,997	+	1,542
*MT-TS1*	5,000	5,068	−	69
*MT-TD*	5,072	5,140	+	69
*MT-CO2*	5,141	5,837	+	697
*MT-TK*	5,829	5,895	+	67
*MT-ATP8*	5,896	6,102	+	207
*MT-ATP6*	6,057	6,737	+	681
*MT-CO3*	6,737	7,520	+	784
*MT-TG*	7,521	7,588	+	68
*MT-ND3*	7,589	7,934	+	346
*MT-TR*	7,935	8,000	+	66
*MT-ND4L*	8,001	8,297	+	297
*MT-ND4*	8,291	9,662	+	1,372
*MT-TH*	9,663	9,731	+	69
*MT-TS2*	9,732	9,790	+	59
*MT-TL1*	9,791	9,861	+	71
*MT-ND5*	9,858	11,669	+	1,812
*MT-ND6*	11,670	12,199	−	530
*MT-TE*	12,200	12,268	−	69
*MT-CYB*	12,273	13,412	+	1,140
*MT-TT*	13,415	13,483	+	69
*MT-TP*	13,487	13,554	−	68
*MT-TF*	14,711	14,780	+	70
*MT-RNR1*	14,781	15,734	+	954
*MT-TV*	15,735	15,803	+	69

* *MT-RNR2* extends from 15,804 to end (16,491) and to 783.

**Figure 1:**
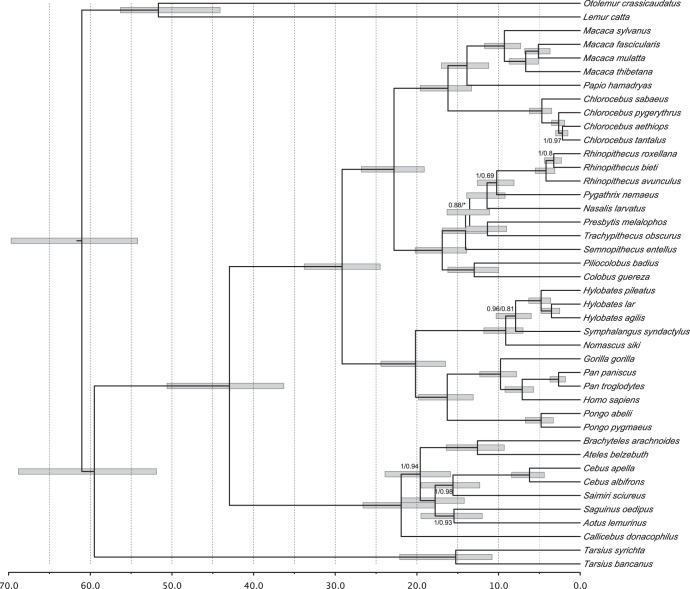
Bayesian phylogeny and timescale of primate evolution inferred from mitochondrial genomes. Nodes were supported by 100% Bayesian posterior probability (PP) and 100% approximate likelihood ratio statistic (aLRT). Exceptions are illustrated by the values of PP/aLRT on nodes. Bars indicate 95% credibility intervals. (*) Node not recovered in ML analysis.

Phylogenetic analyses showed identical Bayesian and ML tree topologies except for the position of *Semnopithecus entellus* as a sister lineage of the ((*Rhinopithecus*, *Pygathrix*), *Nasalis*) clade in ML (Figure 1). Our inferred primate phylogeny based on mitochondrial genomes was similar to the topological arrangement proposed by Perelman et al. [10] although differences were found in the evolutionary affinities of colobine monkeys. With respect to NP, we confirmed that the Pitheciidae represented the first basal offshoot, supporting the sister taxa relationship of Atelidae and Cebidae (100% posterior probability and 0.94 approximate likelihood ratio statistic). Among cebids, *Saimiri* and *Cebus* were arranged as sister groups and *Aotus* formed a monophyletic assemblage with *Saguinus* (a callitrichine), as recently proposed. *Brachyteles* grouped with the Amazonian spider monkey, *Ateles belzebuth*.

The inferred timescale placed the time to the recent common ancestor (TMRCA) of extant neotropical primates in the early Miocene, *ca*. 22 Ma, with a 95% HPD interval ranging from 17.8 to 26.6 Ma, from the late Oligocene to early Miocene (Figure 1). Following the early pitheciid diversification, atelids also separated from cebids in the early Miocene, 19.6 Ma; the credibility interval ranging from the beginning to the end of the early Miocene, 23.9 to 15.9 Ma. The TMRCA of modern cebids was inferred as 17.8 Ma, also in the early Miocene while the *Aotus*/callitrichinae and *Cebus*/*Saimiri* splits were estimated to have taken place in the transition period from the early to middle Miocene (*ca*. 16 Ma). Within *Cebus*, the gracile and robust groups diverged at approximately 6 Ma. Finally, the TMRCA of Atelinae, the splitting between *Ateles* from the *Brachyteles*/*Lagothrix* common stock, was estimated to have occurred 12.6 Ma, from middle to late Miocene. The 95% credibility interval encompasses the whole middle Miocene transition, from 9.3 to 16.4 Ma.

**Figure 2:**
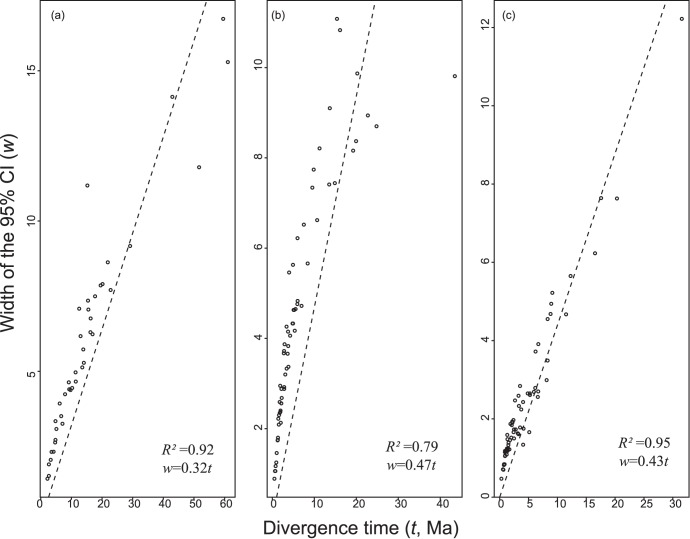
Plots of width of 95% Bayesian credibility intervals (CI, *w*) against inferred divergence times (*t*) with linear regression shown as dashed line. All regressions were significant at *p*<0.01. (a) Mitochondrial genomes; (b) Neotropical primate data reported by Perelman et al [10]; Catarrhine data reported by Perelman et al [10].

The plot of the credibility interval against divergence time estimates from mitochondrial genomes showed a significant fit to the linear regression line *w* = 0.32*t* (*R* ^2^ = 0.92, *p*<0.01) (Figure 2*a*), indicating that, for each 10 Ma of age estimates, the width of the credibility interval approximately increased by 3.2 Ma. Although significant, the fit to the regression line was below 95%, pointing that improvement of divergence time estimates might be achieved by increasing amounts of sequence data, using additional informative calibration priors or both.

Analyses of NP data reported by Perelman et al. [10] showed that the width of credibility intervals and divergence times were associated, with a coefficient equal to 0.47 (*R* ^2^ = 0.79, *p*<0.01) (Figure 2*b*) showing that, for every 10 Ma, the uncertainty associated with the time estimates increased by 4.7 Ma. For catarrhines, the angular coefficient equaled 0.44, but the regression line showed a better fit (*R* ^2^ = 0.95, *p*<0.01) (Figure 2*c*).

## Discussion

The NP timescale estimated from mitochondrial genomes was similar to recent findings using nuclear [25] or combined [26] phylogenomic datasets. This was also the case of the latest extensive analysis of Perelman et al. [10] placing the TMRCA of extant NP at the late Oligocene (25 Ma). Moreover, the age of the Atelidae/Cebidae separation, as well as the TMRCA of modern cebids, were also estimated to have occurred in the early Miocene. Likewise, the separation of *Cebus*/*Saimiri*, *Aotus*/callitrichinae and gracile from robust *Cebus* groups were all in agreement with estimates using mitochondrial genomes.

These similarities show that mitochondrial genomes provide useful data to study primate evolution. Moreover, an assessment of the analyses carried out for NP by Perelman et al. [10] showed that the precision of their estimates was actually lower than those observed in this study. This discrepancy might result from the selected set of calibration priors, although the low *R* ^2^ estimate of Perelman et al. [10] indicated that informative sequence data were not saturated, and that the NP timescale might be improved by including additional genes in analyses. It should be noted that a different scenario was apparent when analyzing the catarrhine data reported by Perelman et al. [10] because the regression line between *w* and *t* showed a better fit (*R* ^2^ = 0.95), indicating that increasing the number of genes may not improve the precision of estimates. This can only be achieved with better calibration priors.

In conclusion, mitochondrial genomes were shown to be as effective as nuclear supermatrices for dating the diversification of extant NP lineages. Nevertheless, it is evident that available data are still far below the ideal theoretical limit, and that sequencing of additional genomes will reduce uncertainties associated with the evolution of the major NP lineages.
